# YOLOv8‐based framework for medial temporal lobe atrophy grading

**DOI:** 10.1002/alz70856_096669

**Published:** 2025-12-24

**Authors:** Tianqing Deng, Yang Lü

**Affiliations:** ^1^ The First Affiliated Hospital of Chongqing Medical University, Chongqing, China; ^2^ The First Affiliated Hospital of Chongqing Medical University, Chongqing, Chongqing, China

## Abstract

**Background:**

Alzheimer's disease (AD) is typically associated with medial temporal lobe atrophy (MTA), and accurate assessment of the degree of MTA is crucial for early diagnosis and disease progression evaluation. The most widely used tool for MTA assessment is the scale proposed by Scheltens et al. in 1992, known for its speed, simplicity, cost‐effective, and clinical universality. However, subjective assessments are often influenced by the evaluator's experience. This study aims to eliminate inter‐rater variability by utilizing artificial intelligence algorithms for automated MTA grading.

**Methods:**

A total of 1524 cases from the ADNI database and local memory clinic were included. Experienced physicians graded MTA from 0 ‐ 4 on coronal MRI by assessing the width of the choroidal fissure, temporal horn, and hippocampal height according to the MTA scale. An automated MTA grading tool based on the YOLOv8 architecture, incorporating the EfficientViT backbone network and a customized adaptive sliding loss, was developed and trained. The performance of this tool was compared with manual grading and other deep learning methods.

**Results:**

The intraclass correlation coefficient between the YOLOv8‐based automated MTA grading and manual grading was 0.852 (95% CI: 0.809‐0.886, *p* < 0.001). The accuracy of the proposed tool was 0.795, outperforming YOLOv5 (0.750), Faster R‐CNN (0.732), Mask R‐CNN (0.755), and U‐Net (0.788) in classification performance on both the ADNI and local memory clinic datasets.

**Conclusion:**

This study demonstrates that the YOLOv8‐based automated MTA grading tool can accurately identify and segment the medial temporal lobe and assess its degree of atrophy. The model also exhibits strong generalizability, which is beneficial for promoting the objective and efficient application of the MTA grading scale in AD diagnosis.

